# Editorial: Novel therapeutic approaches for biliary tract cancer and hepatocellular carcinoma

**DOI:** 10.3389/fcell.2023.1320084

**Published:** 2023-11-03

**Authors:** Dino Bekric, Maria Lina Tornesello, Matthias Ocker, Christian Mayr, Tobias Kiesslich, Daniel Neureiter

**Affiliations:** ^1^ Center of Physiology, Pathophysiology and Biophysics, Institute of Physiology and Pathophysiology Salzburg, Paracelsus Medical University, Salzburg, Austria; ^2^ Cancer Cluster Salzburg, Salzburg, Austria; ^3^ Molecular Biology and Viral Oncology Unit, Istituto Nazionale Tumori IRCCS “Fondazione G. Pascale”, Naples, Italy; ^4^ Medical Department, Division of Hematology, Oncology, and Cancer Immunology, Campus Charité Mitte, Charité University Medicine Berlin, Germany and Tacalyx GmbH, Berlin, Germany; ^5^ Institute of Pathology, University Clinics Salzburg, Paracelsus Medical University, Salzburg, Austria

**Keywords:** hepatocellular carcinoma, biliary tract cancer, hepatobiliary cancer, chemoresistance, therapeutic targets, immunotherapy

## Background

Hepatocellular carcinoma (HCC) and biliary tract cancers (BTC) represent the two major forms of primary liver cancers. Despite the growing efforts to translate the increasing knowledge on molecular alterations of these cancers into treatment options for patients, the actual clinical outcomes remain unsatisfying (Llovet et al., 2021; Valle et al., 2021).

BTCs are fatal gastrointestinal cancers with very poor 5-year survival rates (Zhu et al., 2010). The incidence rates vary across geographic regions: in the Western World, the incidence ranges from 0.5 to 2 per 100,000 population, while in the Eastern World, the incidence is higher at 60 per 100,000 population (Valle et al., 2021). The molecular background of BTC development and progression is complex and remains only partially understood, although it is clear that besides mutational events and dysregulated signaling pathways, aberrant epigenetics also play a role (Mayr et al., 2015; Mayr et al., 2021; Bekric et al., 2023). Possible explanations for the low survival rates include diagnosis at an advanced stage and the development of resistance to, and ineffectiveness of current therapies as well as not standardized second-line therapies for advanced BTC (Rakic et al., 2014; Moik et al., 2019). Non-specific symptoms such as abdominal pain, unexplained weight loss and painless jaundice can lead to late diagnosis and ineffective clinical management (Nagorney et al., 1993). In addition, current therapies, which include radiotherapy, FGFR inhibitors, immunotherapies, combination chemotherapies such as cisplatin and gemcitabine and palliative care, are not able to significantly improve the low median survival (Valle et al., 2010; Rakic et al., 2014).

HCC is the most common form of liver cancer (Sung et al., 2021). This deadly malignancy was responsible for more than 830,000 deaths around the world in 2020 (Sung et al., 2021). HCC is therefore the second leading cause of cancer related mortality globally (McGlynn et al., 2021). Similar to BTC, patients with HCC are usually diagnosed in mid-to-late stages due to unspecified symptoms such as fatigue, nausea, vomiting and abdominal pain, which make successful surgical treatment difficult (Llovet et al., 2021). Patients with advanced-stage HCC are currently treated with immunotherapy in combination with bevaciuzumab, adjuvant chemotherapy after surgery or multikinase/tyrosinkinase inhibitors such as sorafenib and regorafenib (Llovet et al., 2008; Bruix et al., 2015). Although sorafenib treatment improves survival in HCC patients, recent studies report increasing resistance to this multikinase inhibitor (Chen et al., 2015; Keating, 2017). Therefore, differential combinatorial treatment strategies using signaling, epigenetic and immune targets in HCC will be a promising approach to increase therapeutic success in the future (Neureiter et al., 2019; Ocker et al., 2021).

Due to the ineffectiveness and development of resistance to current therapies, the need to identify and provide alternative therapeutic approaches is of paramount importance to alleviate the suffering of BTC and HCC patients. Therefore, the current Research Topic provided a structural platform to identify mechanisms of resistance, novel relevant therapeutic targets and prognostic/predictive markers as well as to demonstrate promising innovative treatment options. The call for papers attracted an astonishing number of 10 highly interesting publications and over 65 authors contributed to this Research Topic in the form of three structured reviews (Guo et al.; Shen et al.; Yang et al.) and seven original research papers (Liu et al.; Wu et al.; Jansson et al.; Liang et al.; Nagashima et al.; Yang et al.; Tang et al.).

This Research Topic covers a variety of subject areas: promising new survival markers after surgery for BTC patients (Jansson et al.), non-invasive preoperative prediction of angiogenesis related markers in BTC (Liu et al.), optimizing strategies for immunotherapy and the current status of adjuvant therapies in HCC are discussed (Liang et al.; Tang et al.; Shen et al.; Guo et al.), characteristics of extrahepatic cholangiocarcinoma (eCCA) are analyzed (Yang et al.; Nagashima et al.), and novel therapeutic strategies for BTC are demonstrated. (Wu et al.; Yang et al.).

We will discuss the highlights of these published manuscripts in short:

## Prognosis and prediction factors in BTC

Improving patient outcomes in BTC requires identifying predictive and prognostic factors.

High levels of vascular endothelial growth factor (VEGF) expression and microvessel density (MVD) correlate with tumor progression and poor prognosis in eCCA patients (Möbius et al., 2007; Thelen et al., 2008; Dongqing et al., 2019). However, current methods for detecting these factors are invasive and challenging to replicate. Liu et al. developed a machine learning tool using regression and classification models for predicting VEGF expression and MVD in eCCA. The MRI-based tool accurately predicted these markers non-invasively in a cohort of 100 BTC patients (Liu et al.).

BTC patients typically experience cancer recurrence within 5 years of surgery, but prognostic factors such as lymph node metastasis and tumor grading can only be observed after tumor resection (Mavros et al., 2014; Koerkamp et al., 2015; Margonis et al., 2016; Bird et al., 2018; Vega et al., 2021). In Jansson et al.'s study, three preoperative immunologic plasma markers were identified - CSF1, TIE2, and TRAIL - that predict survival after surgery in a cohort of 102 BTC patients utilizing high-throughput multiplex immunoassay. CSF1 and TIE2 were found to be negative prognostic factors in BTC, while TRAIL was demonstrated to be a positive prognostic factor (Jansson et al.).

## Immunotherapy in HCC

Immunotherapy is a therapeutic option for HCC patients; however, the immune microenvironment of many tumors suppresses the effectiveness of this treatment (Shen et al.).

To address this Research Topic, Shen et al. provide a valuable review article on HDAC inhibitors in HCC. This article demonstrates the ability of HDACs to improve the effectiveness of immunotherapies in cancer treatment. This can be achieved through increased expression of PD-L1 or the recruitment of NK cells and T cells (Shen et al.).

Liang et al. also identified an efficacy enhancement of immunotherapy in HCC by combining anti-PD-1 antibodies with Abrine, an inhibitor of indoleamine 2,3-dioxygenase 1 (IDO1). In their study, Liang et al. showed that IDO1 is upregulated in HCC cells and can lead to tumor immune escape. They found that using Abrine along with anti-PD-1 antibodies can inhibit immune escape and increase CD8^+^ T cell infiltration, leading to a stronger immune response and anti-tumor effect (Liang et al.).

Taken together, this Research Topic provides insight into the latest efforts to overcome resistance mechanisms of current therapies, discover novel prognostic and predictive markers, and identify alternative anti-BTC/HCC strategies.

We sincerely thank all the authors for their valuable contributions to this Research Topic.
